# Screening candidate microRNAs (miRNAs) in different lambskin hair follicles in Hu sheep

**DOI:** 10.1371/journal.pone.0176532

**Published:** 2017-05-02

**Authors:** Wen Gao, Wei Sun, Jinfeng Yin, Xiaoyang Lv, Jianjun Bao, Jiarui Yu, Lihong Wang, Chengyan Jin, Liang Hu

**Affiliations:** College of Animal Science and Technology, Yangzhou University, Yangzhou, China; China Agricultural University, CHINA

## Abstract

Hu sheep lambskin is a unique white lambskin from China that exhibits three types of flower patterns, including small waves, medium waves, and large waves, with small waves considered the best quality. However, our understanding of the molecular mechanism underlying flower pattern formation in Hu sheep lambskin is limited. The aim of the present study was to further explore the relevance between candidate microRNAs (miRNAs) and developmental characteristics of hair follicles and screen miRNAs for later functional validation. Herein, we employed Illumina Hiseq 2500 to identify differentially expressed miRNAs in hair follicles of different flower patterns with small, medium, and large waves to construct a comprehensive sequence database on the mechanism of hair follicle development. Paraffin sections of lambskin tissue were prepared to assess the structure of different hair follicles. Expression levels of candidate miRNAs in different flower patterns were analyzed by relative quantitation using real-time PCR, combined with histological observation and micro-observation technologies, and the correlation between expression levels of candidate miRNAs and histological properties of hair follicles was analyzed by using SPSS 17.0. A total of 522 differentially expressed miRNAs were identified, and RNA-seq analysis detected 7,266 target genes in different groups of flower patterns. Gene ontological analysis indicated these target genes were mainly involved in cell proliferation, differentiation, growth, apoptosis, and ion transport, and 14 miRNAs, including miR-143, miR-10a, and let-7 were screened as candidate miRNAs in Hu sheep hair follicle growth and development. In the same field of vision, variance analysis showed that the number of secondary follicles in small waves was significantly larger than that in large and medium waves (*P*<0.01); the diameter of the primary and secondary follicles in large waves was respectively larger than those in medium and small waves (*P*<0.01). Combined with correlation analysis between miRNA expression and histological properties of hair follicles, highly significant differences in miRNA-143 expression levels between large and small waves were observed (P<0.01), and significant differences in the miRNA-10a expression levels between large and small waves (*P*<0.05) and in let-7i expression levels between large and medium waves were observed (*P*<0.05). Significant differences in the expression of novel miRNAs of NW_004080184.1_6326 between medium and large waves were detected (P<0.05), and highly significant differences between medium and small waves were observed (P<0.01). Highly significant differences in the expression level of NW_004080165.1_8572 between medium and large and small waves (*P*<0.01), in that of NW_004080181.1_3961 between medium and small waves (*P*<0.01), and in that of NW_004080190.1_13733 between medium and large waves were observed, whereas no significant differences in the other miRNAs among large, medium, and small waves were detected. Overall, the present study showed that miRNA-143, miRNA-10a, let-7i, NW_004080184.1_6326, NW_004080165.1_8572, NW_004080181.1_3961, and NW_004080190.1_13733 could be considered as important candidate genes, indicating these seven miRNAs may play significant roles in hair follicle growth and development in Hu sheep lambskin.

## Introduction

Hu sheep is a unique breed of white lambskin sheep that has been domesticated in China for about 800 years. Hu sheep have several excellent characters such as good adaptability, high prolificacy and reproduction, early maturation, and high lactation. Furthermore, the value of Hu sheep lambskin in the international market is relatively high and this is mainly due to its superior quality, spotless white color, silky smooth texture, and peculiar flower patterns that are similar to water waves. Based on the width of the flower pattern, Hu sheep lambskin can be divided into three wave types, namely, large, medium, and small, with the small waves showing the best quality [1]. Several factors determine the type of lambskin pattern, which include fineness, density, and curvature of wool. The hair follicle is an accessory organ of the skin that has complex morphology and structure. Hair follicles control the growth of hair and can be divided into primary follicle and secondary follicle, which respectively grow myelinated hair and unmyelinated hair [2]. Therefore, studying the inheritance pattern of hair follicles may reveal the regulatory mechanism of flower pattern formation in Hu sheep lambskin at the molecular level, which in turn may be applied in breeding schemes for the improvement of Hu sheep lambskin quality.

Current research studies on miRNAs mainly focus on muscle, liver, ovary and cancer tissues, whereas most of studies on sheep hair follicles concentrate on its growth cycle and only a few studies centered on miRNA expression and hair follicle development in Hu sheep lambskin. He et al. [3] extracted the total RNA from alpaca skin, prepared miRNA chips and screened miRNAs in the ear and back skin by using Affymetrix miRNA chip interspecies hybridization. Andl et al. [4] reported that Dicer mRNA and miRNA were expressed in the skin tissue of mice, indicating that miRNA plays an important role in the development of skin and hair follicle. Zhu et al. [5] found that the key gene of chromogenesis, *Mitf*, could be inhibited by transducing the lentiviral vector of miR-25 into the melanophores of alpaca skin, prompting them to hypothesize that miR-25 inhibits *Mitf* expression, and miR-25 possibly participates in the regulation of hair color. Mardaryev et al. [6] found that the expression of miR-31 significantly increased during skin and hair follicle growth. Furthermore, they found that miR-31 plays a significant role in regulating hair growth and hair follicle development in mice through bone morphogenetic protein (BMP) and Wingless-Int (WNT) signaling pathways. In addition, Tang et al. [7] found that miR-31 is highly expressed during hair follicle growth and downregulated in telogen.

To date, most investigations have focused on polyembryony and meat performance instead of lambskin quality, thereby hindering further improvements in breeding Hu sheep with higher quality lambskin and in protecting germplasm resources. A number of histological assessment of Hu sheep lambskin have been performed; however, our understanding of the specific molecular mechanism underlying the growth and development of hair follicles and the formation of different flower patterns in Hu sheep lambskin is limited. In the present study, high-throughput sequencing and bioinformatics analysis were initially employed to identify differentially expressed miRNAs that influence the growth and development of hair follicles as well as the formation of specific flower patterns in Hu sheep lambskin. By combining histological observation and the micro-observation technology, the correlation between the expression of 14 miRNAs in different wave patterns and the histological properties of hair follicles was analyzed. Our results showed that seven candidate miRNAs, namely, miRNA-143, miRNA-10a, let-7i, NW_004080184.1_6326, NW_004080165.1_8572, NW_004080181.1_3961, and NW_004080190.1_13733, are involved in the development of Hu sheep lambskin hair follicles, which in turn may facilitate in the elucidation of the molecular mechanism underlying its growth and development in Hu sheep lambskin.

## Materials and methods

### Ethics statement

This study was conducted in strict compliance with the recommendations of the Guide for the Care and Use of Laboratory Animals of Jiangsu Province and of the Animal Care and Use Committee of the Chinese Ministry of Agriculture. The government of Jiangsu Province (Permit Number 45) and the Ministry of Agriculture of China (Permit Number 39) approved the protocol performed in this study. All efforts were made to minimize animal suffering.

### Experimental animals

Six pairs of full-sib Hu sheep were selected at birth at the Suzhou stud farm in China. Each pair consisted of one individual with predominantly large-wave wool, one with medium-wave wool, and one with predominantly small-wave wool. To extract RNA from the hair follicles, about 1 cm of the hair root was excised and placed into a microtube that contained with 1 mL of TRIzol^®^ Reagent (Invitrogen, Carlsbad, CA, USA) and surrounded by drikold. The quantity of hair root was collected up to a third of the volume of the microtube. Each individual was locally anaesthetized by subcutaneous injection of 0.1 mL of 2% procaine (Changzhou Sunchem Pharmaceutical Chemical Material Co., Ltd, Changzhou, China) prior to the removal of the back skin tissue at an approximate size of 1.5 cm^2^. Then, the back skin tissue was flattened on the cardboard, which was then immersed in 4% paraformaldehyde solution (Sinopharm Chemical Reagent Co., Ltd, Shanghai, China) in a centrifuge tube.

### Total RNA extraction and small RNA library preparation

Total RNA was extracted from the hair roots of six pairs of full-sib individuals using a buffer containing ß-mercaptoethanol and guanidine that was provided in an RNAiso Plus kit in accordance with the manufacturer’s instructions (Takara Biotechnology Dalian, Co. Ltd., China). RNA was eluted with 40 uL of RNase-free water, and the quantity and purity of total RNA were measured by using a NanoDrop ND-1000 spectrophotometer (NanoDrop Technologies, Wilmington, DE, U.S.). Samples with purity (A260/A280) of 1.8–2.0 were used in the subsequent experiment. Total RNA was purified using an RNAclean Kit (TIANGEN BIOTECH Co., Ltd, Beijing, China). The samples for small RNA sequencing were prepared in accordance with the instructions that were provided by the manufacturer of the Illumina sequencing kit.

### Small RNA sequencing and processing of reads

Small RNA libraries were sequenced on an Illumina HiSeq 2500 system with 50-base pair (bp) single-end reads according to the manufacturer’s protocol. Then, a series of treatments, including quality inspection of bases and selection of length, as well as sequencing fragments of reliable quality were performed, which was followed by estimation of varieties and quantities by using a distribution statistic. The length range of small RNAs is 18–30 nt and that of miRNAs is 21–22 nt, the peak of the length distribution can help to determine small RNA species. All small sequencing data have been uploaded to the Sequence Read Archive database (SRR3099013, SRR3099014, and SRR3099015).

### Validation of small RNA sequencing by real-time PCR

Differentially expressed miRNAs, namely, miRNA-10a, miR-199a-3p, let-7i, NW_004080190.1_13733, and NW_004080166.1_9139 were randomly selected for real-time PCR, and the U6 gene was used as reference (S1 Table). The SYBR Green I method was used for quantitative testing to verify the reliability of the sequencing results. A standard curve was established using a cDNA gradient dilution, and three replicates were prepared for each sample. The relative expression of the target gene was calculated by using the following formula: Relative expression = 2^-ΔΔCt^, ΔΔCt = (C_target gene_—C_housekeeping gene_)_(large waves, medium waves)_—(C_target gene_—C_housekeeping gene_)_small waves_.

### Paraffin section fabrication

The lambskin tissues were treated according to the conventional paraffin section method. After dehydration, transparent, embedding, sectioning, and HE staining, the structure of the hair follicles was observed under a microscope and representative images were captured. Three different fields of view of each section were selected. The number and diameter of the primary and secondary hair follicles were respectively determined. Each hair follicle feature was compared among the large, medium, and small waves by using ANOVA.

### Quantitation of 14 miRNAs by real-time PCR

Total RNA was extracted by using a buffer that contained β-mercaptoethanol and guanidine, which was provided in an RNAiso Plus Kit, following the manufacturer’s instructions (Takara Biotechnology Dalian, Co., Ltd.). RNA was eluted with 40 μL of RNase-free water. The concentration of RNA was measured by using a NanoDrop ND-1000 spectrophotometer (NanoDrop Technologies, Wilmington, DE, USA). Samples with purity (A_260_/A_280_) of > 1.8 were used. A total of 2 μg of RNA from each sample was transcribed into cDNA by using a TaKaRa reserve transcription kit (TaKaRa Biotechnology Dalian, Co., Ltd.) according to manufacturer’s instructions. The primers used in real-time PCR are listed in S1 Table.

The U6 gene was employed as reference, and the relative expression level of the target gene was calculated by using 2^-ΔΔCt^ method. According to a TIANGEN miRcute miRNA fluorescence quantitative detection kit, the optional reaction system was a 20-μL reaction containing 10 μL of 2× miRcute miRNA Premix, 0.4 μL of the forward primer, 0.4 μL of the reverse primer, 1 μL of the first-strand cDNA of the miRNA, and 8.2 μL of ddH2O. The PCR conditions included 40 cycles of 2 min at 94°C, 20 s at 94°C, and 34 s at 60°C.

### Statistical analysis

SPSS17.0 was used to calculate the Ct values and standard errors among repeat samples, and the difference in relative gene expression levels was analyzed by using the 2^-ΔΔCt^ method. The relative expression of the target gene was calculated by using the following formula: Relative expression = 2^-ΔΔCt^, ΔΔC t = (C_target gene_—C_housekeeping gene_)_(large waves, medium waves)_−(Ctarget gene—C_housekeeping gene_)_(small waves)_. The expression levels of miRNA in different wave patterns were compared using ANOVA.

## Results

### miRNA sequencing and assembly

Three kinds of waves (large, medium, and small) were selected for analysis. The miRNA library was established using a Illumina Hiseq 2500 system, and sequencing generated 7,964,087, 7,167,843, and 7,534,406 miRNAs from large, medium, and small wave wool, respectively. Detailed results of the sequencing and assembly are shown in Table 1.

**Table 1:** Summary of Illumina sequencing and miRNA assembly.

Sample name	Total reads	Low-quality reads	Clean reads	Clean read (%)
Large	7964087	998345	6965742	87.46
Medium	7167843	2197329	4970514	69.34
Small	7534406	2174488	5359918	71.14

### Differential expression analysis of miRNAs

**Fig 1:**
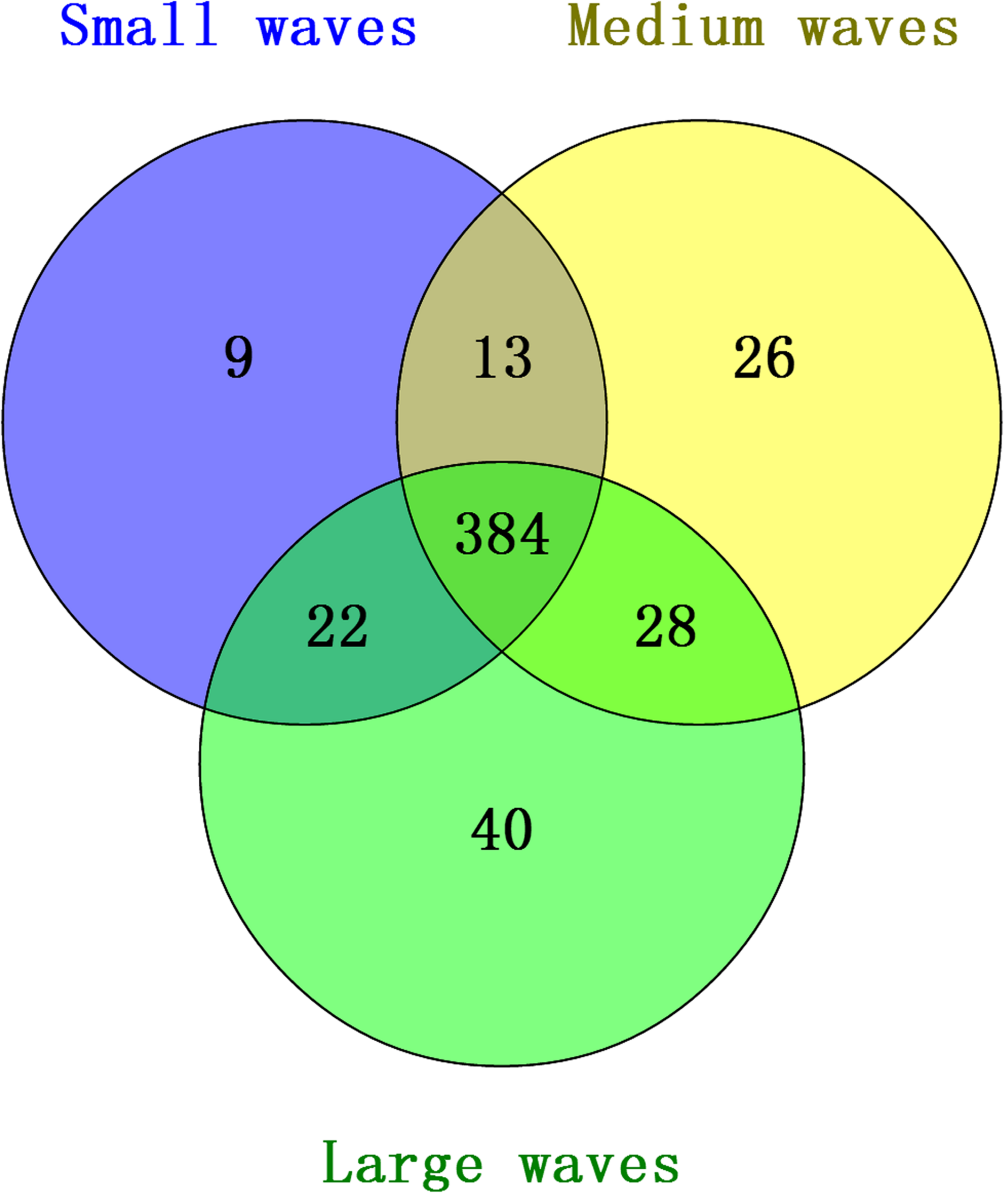
The distribution of miRNA in small waves, medium waves and large waves.

A total of 522 differentially expressed miRNAs were screened using high-throughput sequencing. Of these miRNAs, 384 were co-expressed in the three patterns, whereas 9, 26, and 40 miRNAs were expressed in small waves, medium waves, and large waves, respectively (Fig 1). Statistical analysis was employed to identify significant differentially expressed miRNAs in the two kinds of patterns. The results showed that there were 36 differentially expressed miRNAs, including 29 novel miRNAs. Among these miRNAs, 10, 10, and 5 miRNAs were differentially expressed in small and large, small and medium, and medium and large waves, respectively, whereas no differentially expressed miRNAs in three kinds of waves were detected.

### Predictive analysis of target genes of differentially expressed miRNAs

The target genes of 522 differentially expressed miRNAs were predicted by using the miRanda predict software to annotate their biological functions. The result showed that there were 1,008, 2,062, and 4,169 target genes in small and large, medium and large, and small and medium waves, respectively (S2–S4 Tables).

### Functional analysis of target genes

Gene ontology (GO) and pathway analyses were performed for these target genes. GO analysis identified 306 GO terms in small and large waves (Table 2). There were 217 GO terms in small and medium waves (Table 3). Finally, there were 371 GO terms in medium and large waves (Table 4). These were mainly involved in cell process, metabolism, ion transport, growth, and development. Pathway analysis showed that the target genes were related to 53, 61, and 65 signal pathways in small and large, medium and large, small waves and medium waves, respectively (Tables 5–7), including metabolic pathways, ion transport, cell proliferation and differentiation, and cell adhesion.

**Table 2:** The top 10 enriched GO terms of target genes for differentially expressed miRNAs between small and large waves.

GO_id	GO_term	Count	Go category	P-value
GO:0008152	metabolic process	325	biological process	1.83E-03
GO:0071704	organic substance metabolic process	302	biological process	2.09E-04
GO:0044237	cellular metabolic process	301	biological process	1.23E-04
GO:0044238	primary metabolic process	289	biological process	4.57E-04
GO:0043170	macromolecule metabolic process organelle	259	biological process	7.00E-05
GO:0044260	cellular macromolecule metabolic process	247	biological process	1.17E-05
GO:0019222	regulation of metabolic process	204	biological process	4.56E-03
GO:0006807	nitrogen compound metabolic process	195	biological process	5.64E-03
GO:0009058	biosynthetic process	189	biological process	5.55E-07
GO:0031323	regulation of cellular metabolic process	189	biological process	1.25E-04

**Table 3:** The top 10 enriched GO terms of target genes for differentially expressed miRNAs between small and medium waves.

GO_id	GO_term	Count	Go category	P-value
GO:0050794	regulation of cellular process	789	biological process	2.22E-02
GO:0016043	cellular component organization	474	biological process	4.15E-02
GO:0031323	regulation of cellular metabolic process	469	biological process	2.08E-02
GO:0080090	regulation of primary metabolic process	465	biological process	5.97E-03
GO:0051179	localization	456	biological process	3.54E-02
GO:0006793	phosphorus metabolic process	342	biological process	4.67E-03
GO:0030154	cell differentiation	342	biological process	3.41E-02
GO:0006796	phosphate-containing compound metabolic	339	biological process	4.98E-03
GO:0051234	establishment of localization	338	biological process	2.62E-02
GO:0043412	macromolecule modification	330	biological process	1.21E-02

**Table 4:** The top 10 enriched GO terms of target genes for differentially expressed miRNAs between medium and large waves.

GO_id	GO_term	Count	Go category	P-value
GO:0005488	binding	865	molecular function	2.15E-02
GO:0005515	protein binding	817	molecular function	1.19E-02
GO:0065007	biological regulation	605	biological process	2.49E-02
GO:0050789	regulation of biological process	578	biological process	1.47E-02
GO:0050794	regulation of cellular process	544	biological process	6.24E-03
GO:0050896	response to stimulus	436	biological process	2.06E-02
GO:0019222	regulation of metabolic process	372	biological process	1.40E-02
GO:0051716	cellular response to stimulus	356	biological process	1.60E-02
GO:0007154	cell communication	329	biological process	3.58E-03
GO:0005488	binding	865	molecular function	2.15E-02

**Table 5:** The top 10 enriched KEGG pathway of target genes for differentially expressed miRNAs between small and large waves.

Pathway ID	Pathway	Gene counts	P-value
ko01100	Metabolic pathways	76	2.30E-05
ko05200	Pathways in cancer	31	1.23E-04
ko05152	Tuberculosis	24	7.11E-06
ko01110	Biosynthesis of secondary metabolites	21	2.02E-02
ko05166	HTLV-I infection	20	2.56E-02
ko04510	Focal adhesion	18	9.92E-03
ko04910	Insulin signaling pathway	17	2.85E-04
ko04726	Serotonergic synapse	15	1.29E-03
ko01120	Microbial metabolism in diverse environments	14	2.42E-02
ko05168	Herpes simplex infection	14	4.50E-02

**Table 6:** The top 10 enriched KEGG pathway of target genes for differentially expressed miRNAs between small and medium waves.

Pathway_id	Pathway_name	Gene counts		P-value
ko01100	Metabolic pathways		182	3.30E-05
ko04151	PI3K-Akt signaling pathway		70	1.26E-04
ko05200	Pathways in cancer		67	1.80E-04
ko04510	Focal adhesion		54	1.33E-06
ko04810	Regulation of actin cytoskeleton		53	1.67E-05
ko04062	Chemokine signaling pathway		51	1.97E-07
ko04144	Endocytosis		51	1.64E-05
ko05205	Proteoglycans in cancer		48	3.68E-03
ko05203	Viral carcinogenesis		39	3.72E-02
ko04722	Neurotrophin signaling pathway		34	5.60E-04

**Table 7:** The top 10 enriched KEGG pathway of target genes for differentially expressed miRNAs between medium and large waves.

Pathway_id	Pathway_name	Gene counts		P-value
ko01100	Metabolic pathways		122	1.88E-03
ko05200	Pathways in cancer		55	6.99E-06
ko04151	PI3K-Akt signaling pathway		47	3.14E-03
ko04010	MAPK signaling pathway		42	5.20E-04
ko05205	Proteoglycans in cancer		37	1.79E-03
ko04510	Focal adhesion		36	2.14E-04
ko04144	Endocytosis		32	3.63E-03
ko04810	Regulation of actin cytoskeleton		32	7.87E-03
ko04062	Chemokine signaling pathway		30	1.49E-03
ko05202	Transcriptional misregulation in cancer		28	5.06E-04

### Validation of small RNA sequencing by RT-PCR

To confirm the reliability of sequencing results, five miRNAs were selected for RT-PCR verification. The sequencing results were basically identical to that of RT-PCR of differentially expressed miRNAs (Table 8).

**Table 8:** The comparison on the results of sequencing and RT-PCR for differentially expressed miRNAs.

Small RNA sequencing			RT-PCR	
miRNA	groups	Log_2_(FC)	Log_2_(FC)	*P* value
miR-10a	1	1.630	1.352	0.099
	2	-1.179	-0.509	0.017*
	3	-2.809	-2.508	0.789
miR-199a-3p	1	-1.543	-0.654	0.359
	2	-2.900	-3.156	0.002**
	3	-1.613	-0.811	0.694
Let-7i	1	-1.034	-0.851	0.021*
	2	-0.761	-0.436	0.628
	3	0.273	0.699	<0.001**
NW_004080190.1_13733	1	-1.186	-0.697	0.707
	2	-0.285	-0.658	0.177
	3	1.165	0.901	0.043*
NW_004080166.1_9139	1	1.672	1.014	0.021*
	2	-0.313	-0.787	0.294
	3	-1.984	-1.844	0.001**

Note: 1 large and medium waves; 2 large and small waves; 3 medium and small waves.

*P<0.05

**P<0.01

### Histological properties of different patterns of lambskin hair follicles

**Fig 2:**
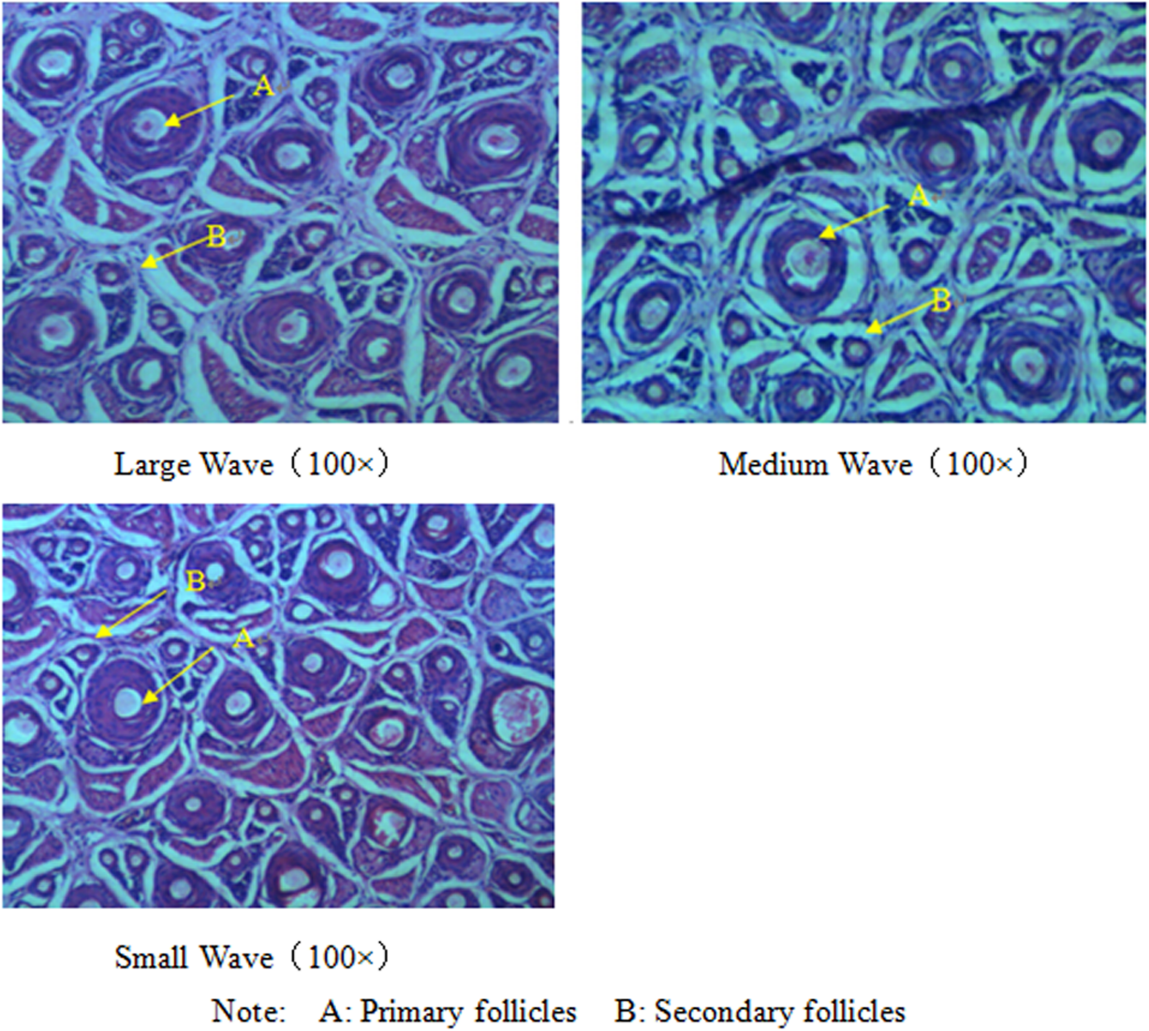
Transverse section images of hair follicles in large, medium and small waves.

Cross-sectional images of skin tissue sections of large, medium, and small wave lambskins of Hu sheep is shown in Fig 2. The statistical results of each indicator of hair follicle are shown in Table 9.

**Table 9:** Histological properties of hair follicles in large, medium and small waves.

Group	Number of individuals	Number of primary follicles	Number of secondary follicles	Diameter of primary follicles	Diameter of secondary follicles
Large waves	6	40.08±1.62a	21.37±0.37A	99.14±1.62A	49.59±0. 94A
Medium waves	6	38.12±0.89a	21.02±0.52A	89.67±1.92B	44.11±0.92B
Small waves	6	37.67±1.75a	29.89±0.49B	87.86±1.60B	44.78±1.34B

Note: Means with different lower-case letters within the same column indicate significant differences between different rows. Means with different capital superscripts within the same column indicate extremely significant differences between different rows. Means with the same lower-case letter within the same column indicate no significant differences between different rows.

The diameter of the primary follicles was larger than that of secondary follicles regardless of large, medium, or small waves. The distribution of hair follicles is mainly in the form of hair follicle groups, centering on primary hair follicles, and secondary hair follicles are located adjacent to the primary hair follicles (Fig 2).

In the same field of view, no significant differences in primary follicles among the large, medium and small wave lambskins were observed (P>0.05); the number of secondary follicles in the small waves was significantly higher than that in large and medium waves (P<0.01), whereas the number of secondary follicles in the medium wave was similar to that in the small waves (P>0.05). Consequently, the total number of hair follicles in the small waves was higher than that in the large and medium waves in the same unit area. The diameters of the primary and secondary follicles in the large waves were significantly larger than those in the medium and small waves (P<0.01), whereas no significant difference was observed between the medium and small waves (P>0.05) (Table 9).

### Expression of the 14 miRNAs in large, medium, and small waves in lambskin hair follicles

The level of expression of miRNA-143 in the large waves was significantly lower than that in the large waves (P<0.01). The expression level of miRNA-10a in the small waves was significantly higher than that in the large and medium waves (P<0.05) and that of let-7i was significantly higher in the medium waves compared to that in the large waves (P<0.05). In the differentially expressed novel miRNAs, the expression level of NW_004080184.1_6326 in medium waves was significantly lower than those in the large and small waves, respectively (P<0.05; P<0.01). The expression of NW_004080165.1_8572 in the medium waves was significantly different compared to that in the large and small waves (P<0.01). NW_004080181.1_3961 was expressed at a higher level in small waves than in medium waves (P<0.01). Highly significant differences in NW_004080190.1_13733 expression was observed between medium and large waves (P<0.01). No significant difference in the expression levels of the rest of the miRNAs were observed among the large, medium, and small waves (Table 10).

**Table 10:** Relative expression quantities of the 14 miRNAs in large, medium and small waves.

Differentially expressed miRNA	Large waves (6 individuals) Relative expression quantities	Medium waves (6 individuals) Relative expression quantities	Small waves (6 individuals) Relative expression quantities
miRNA-143	0.49±0.11A	0.83±0.07AB	1B
miRNA-10a	0.61±0.27a	0.57±0.24a	1b
let-7c	0.92±0.21a	0.97±0.17a	1a
miR-199a-3p	0.87±0.072a	0.92±0.13a	1a
let-7i	0.83±0.14a	1.31±0.08b	1ab
NW_004080189.1_7961	0.87±0.08a	0.81±0.13a	1a
NW_004080184.1_6535	1.09±0.14a	0.92±0.22a	1a
NW_004080184.1_6326	0.74±0.15a	0.57±0.13Aa	1B
NW_004080165.1_8572	1.21±0.12A	0.27±0.18B	1A
NW_004080166.1_9139	1.02±0.09a	1.17±0.11a	1a
NW_004080181.1_3961	0.81±0.07AB	0.23±0.14A	1B
NW_004080174.1_726	1.10±0.14a	0.91±0.08a	1a
NW_004080166.1_10417	1.29±0.17a	1.09±0.13a	1a
NW_004080190.1_13733	0.32±0.82A	1.47±0.41aB	1a

### Correlation analysis between 14 miRNAs expression and histological properties of hair follicles

The association between the expression of 14 miRNAs and each histological properties of hair follicle was analyzed by using SPSS 17.0 (Tables 11–12), which showed that these were involved in the growth and development of hair follicles.

**Table 11:** Correlation between 14 miRNAs expressions and diameters of hair follicle.

miRNA name	Diameter s of primary hair Follicle			Diameters of secondary hair follicle		
	Large waves	Medium waves	Small waves	Large waves	Medium waves	Small waves
miRNA-143	-0.351*	-0.067	0.114	-0.304	-0.215	-0.224
miRNA-10a	0.093	-0.403	-0.412	0.424**	-0.105*	-0.304**
let-7c	0.423	0.109	-0.075*	-0.195*	-0.138*	-0.211
miR-199a-3p	-0.484	-0.435	0.232	0.172	-0.127	-0.510
let-7i	0.243*	-0.217**	0.042	0.302	-0.244	0.299
NW_004080189.1_7961	-0.483*	-0.381	0.673	-0.223**	-0.258	0.391*
NW_004080184.1_6535	-0.017	-0.531*	-0.376*	0.147	-0.172	-0.545
NW_004080184.1_6326	0.496	-0.271	-0.110	0.173*	-0.214**	0.421**
NW_004080165.1_8572	0.026	-0.143	0.304	0.437*	-0.364	-0.464
NW_004080166.1_9139	0.111*	0.324*	0.576	0.266*	-0.217	-0.336
NW_004080181.1_3961	0.391	0.307	0.421	0.417	0.539	0.236
NW_004080174.1_726	0.337	0.573	0.407	0.572	-0.447*	0.215*
NW_004080166.1_10417	-0.180	-0.408	0.180	0.401	-0.331	-0.491*
NW_004080190.1_13733	0.419	-0.221	-0.239	0.306*	-0.311*	-0.337

Note

*P<0.05

**P<0.01

**Table 12:** Correlation between 14 miRNAs expressions and numbers of hair follicle.

miRNA name	Numbers of primary hair Follicle			Numbers of secondary hair follicle		
	Large waves	Medium waves	Small waves	Large waves	Medium waves	Small waves
miRNA-143	-0.167	0.411	-0.450	-0.210**	-0.271	0.395**
miRNA-10a	-0.176	0.264	0.650	-0.323*	-0.264	0.502**
let-7c	-0.781	-0.166	0.271	0.043	0.457	0.468**
miR-199a-3p	-0.298	0.290	0.121	0.539*	0.301	0.273**
let-7i	-0.185	0.407	0.161	-0.121*	0.370*	0.252
NW_004080189.1_7961	-0.025	0.679	0.326	-0.441	-0.221	0.473*
NW_004080184.1_6535	0.311	-0.109	0.494	-0.207	0.623	0.219**
NW_004080184.1_6326	0.087	0.107	0.173	-0.310	-0.111	0.102*
NW_004080165.1_8572	0.477	-0.010	0.664	-0.352	0.114*	0.340**
NW_004080166.1_9139	-0.312	0.435	-0.361	0.042	—0.411	0.522*
NW_004080181.1_3961	0.376	0.234	0.248	0.180	0.336*	0.551*
NW_004080174.1_726	0.389	0.287	0.379	0.456	0.367	0.419
NW_004080166.1_10417	-0.117	0.127	0.121	-0.107	-0.223	0.380*
NW_004080190.1_13733	0.209	-0.176	-0.211	-0.391	0.281	-0.372

Note

*P<0.05

**P<0.01

## Discussion

MiRNAs function by interacting with its target genes. The present study determined that miRNAs could regulate multiple target genes and each target gene could be regulated by multiple miRNAs. The relationship between miRNAs and target genes plays an important role in the regulation of hair follicle growth and pattern formation; therefore, it is of great significance to explore the regulatory mechanisms underlying the growth and development of hair follicles in Hu sheep. Bioinformatics methods are currently employed to predict target genes based on the fact that miRNAs can combine with its target genes; however, some incomplete complementary sequence may also be paired, so there must be some false positives in the generated results.

Andl et al.[4] and Yi et al. [8] demonstrated the main role of miRNAs in mice was to maintain skin hair follicle morphogenesis and development in mice, and this discovery has paved the way for further investigations on the role of miRNAs in hair follicles. Currently, miRNA expression profiling in skin and hair or feather follicles has been employed to identify miRNAs that are related to hair follicle development and growth, particularly in sheep, goat, and duck [9–11]. Liu et al. [12] detected that 316 conserved miRNAs and 22 novel miRNAs in the skin of cashmere goats by using Illumina’s Solexa sequencing technology. Wu et al. [13] identified 205 conserved miRNAs and 9 novel miRNAs by RNA-Seq in goat hair follicles. In the present study, we found 522 differentially expressed miRNAs in the hair follicles of different patterns. These differences in results may various among breeds and reference genomes.

Hair follicle development and its postnatal regeneration are characterized by dramatic changes in its microanatomy and cellular activity, which are controlled by multiple signaling pathways, transcription factors, and epigenetic regulators, including miRNAs [14]. Some researches that are based on gene regulatory networks of hair follicles showed that the Wnt [15], transforming growth factor-β (TGF-β) [16], mitogen-activated protein kinase (MAPK) [17], sonic hedgehog (Shh) [18], BMP [19], Notch, and JAK-STAT [20] signaling pathways widely participate in hair follicle development, morphogenesis, and hair follicle cycling. The Wnt signaling pathway stimulates hair follicle stem cells to transformation from telogen to growth. Yang et al. [21] showed that miRNA-148b activates the Wnt/nt/d via miRNA-148bs that activate follicle stem cells to promote the transformation of hair follicles. In most situations, MAPK signaling pathway usually plays a major role as a performer, and TGF-β signaling pathway is a leader to activate other signaling pathways, and Shh, Notch, and JAK-STAT signaling pathways play a fine-tuning role. Pathway analysis has indicated that the target genes are involved with 53, 65, and 61 signal pathways in small and large, medium and large, small and medium waves, respectively, including the metabolic, cancer, MAPK signaling, and PI3K-Akt signaling pathways. These pathways involve cell proliferation and differentiation, cell growth, and development, and thus these differentially expressed miRNAs and target genes may participate in hair follicle development.

The present study screened 36 differentially expressed miRNAs, including 29 novel miRNAs and 7 known miRNAs, although no differentially expressed miRNAs in the three patterns were observed. In these miRNAs, miRNA-143 was upregulated in hair follicles and is differentially expressed in small and large waves. No studies on the function of miRNA-143 in hair follicles have been conducted, although the results of the present study suggest that it might play an important role in the growth and development of hair follicles. Previous studies have shown that miRNA-143 plays an important role various malignancies [22–23]. miRNA-143 has been reported to be downregulated in gastric, lung, cervical, and bladder cancer. The upregulation of miR-143 in these malignancies inhibits cell growth, which in turn indicates that it participates in cell differentiation, proliferation, apoptosis, and metabolism [24–28]. In the present study, we found that LIM-only protein 4 (*LMO4*) was the direct target gene of miRNA-143 and NW_004080184.1_632. Lu et al. [29] reported that *LMO4* interacts with Smad proteins and increases the effect of TGFβ on epithelial cell growth.What’s more, it was found that *LMO4* was upregulated in epithelial cells, particularly during active mesenchymal-epithelial interactions, including the epidermis, hair follicles, and mammary gland [30–33]. let-7c and let-7i belong to the let-7 family, which is an important tumor-suppressor miRNA, and both are downregulated. These regulate cell cycle-related genes and participate in cell apoptosis [34–35]. miR-199a-3p is involved in cell proliferation and development. In liver cancer, miR-199a-3p inhibits the growth of hepatoma cells [36], and in testicular cancer, it reduces tumor cell proliferation, migration, and infection [37]. In addition, miR-199a-3p also participates in cell differentiation of C2C12 myoblasts. miR-199a-3p is the most widely reported in AKT/mTOR signaling pathway, which plays an important role in cell signaling and regulates various biological processes such as cell proliferation, metabolism, growth, differentiation, and apoptosis. Shen et al. [38] showed that miR-199a-3p suppresses the AKT/mTOR signaling pathway and cell proliferation *in vitro*. It has also been found that miR-10a participates in cell proliferation and migration, whereas in contrast to miR-199a-3p, miR-10a promotes tumor cell proliferation, invasion, and migration [39–40]. Fibroblast growth factor-1 (*FGF1*), the target gene of NW_004080189.1_7961, is a member of the fibroblast growth factor (FGF) family. *FGF1* is involved in the growth and development of various tissues and organs and can significantly promote angiogenesis and cell division. *FGF1* also plays a significant role in cell proliferation, migration, and differentiation [41–42]. Previous studies have shown that *MT4* promotes tumor growth and metastasis and influences cell proliferation. Pave et al. [43] reported that *MT4* promotes cancer cell proliferation *in vivo* and *in vitro* by promoting an outside-in signaling through the EGFR pathway. Based on this information, the differentially miRNAs evaluated in the present study may be involved in cell proliferation, differentiation, and apoptosis, as well as the growth and development of hair follicles in Hu sheep lambskin. In addition, nine highly and differentially expressed novel miRNAs (NW_004080189.1_7961, NW_004080184.1_6535, NW_004080184.1_6326, NW_004080165.1_8572, NW_004080166.1_9139, NW_004080181.1_3961, NW_004080174.1_726, NW_004080166.1_10417, and NW_004080190.1_13733) were screened and may also play important roles in the growth and development of hair follicles of Hu sheep.

To study the effect of 14 differentially expressed miRNAs on different wave patterns in Hu sheep lambskin, association analysis was employed to examine the miRNA expression in different wave patterns and histological properties of hair follicle such as the diameter and number of primary and secondary follicles. Seven miRNAs were differentially expressed in different wave patterns, namely, miRNA-143, miRNA-10a, and let-7i, and four novel miRNAs, including NW_004080184.1_6326, NW_004080165.1_8572, NW_004080181.1_3961, and NW_004080190.1_13733. Correlation analysis indicated that these 7 miRNAs could be considered as candidate miRNAs for the growth and development of hair follicles in Hu sheep lambskin. In the present study, miRNA-143 was one of the upregulated miRNAs in Hu sheep hair follicular tissues. Additionally, the results showed that the expression level of miRNA-143 in small waves was higher than that in large and medium waves at the same period, and highly significant differences between large and small wave lambskin hair follicles were observed. Consequently, we speculated that miRNA-143 regulates sheep hair growth during hair follicle cell development. Research studies on miRNA -10a, let-7c, and let-7i are mainly related to cancer and their roles in animal hair follicle development are largely unknown. The role of miRNA-10a in tumors is to promote tumor cell proliferation and differentiation [44–45]. Conversely, as an important tumor suppressor, the miRNAs in let7 families are downregulated in tumor cells and are involved in apoptosis [34,35]. Members of the let7 family regulate the formation of skin melanoma [46]. The essential functions of members of the let7 family in goat and sheep skin have been previously investigated [47]. Expressed in both anagen and telogen of hair follicles in cashmere goat, miRNA-1et7a plays a major role in regulating hair follicle development [48]. In the present study, miRNA expression in medium and small waves were significantly different, which was similar to that of let-7i expression in large and medium waves. These observations suggest that the differential expression of miRNA-10a and let-7i in different pattern lambskin hair follicles influences the growth and development of Hu sheep hair follicles.

In novel miRNAs, NW_004080184.1_6326 was significantly upregulated (P<0.01) in small and large waves, whereas it was downregulated in medium waves. The expression level of NW_004080165.1_8572 in large and small waves was higher than that in medium waves (P<0.01). Significant differences in the expression level of NW_004080181.1_3961 were observed between medium and small waves, as well as in NW_004080190.1_13733 expression between large and medium waves. Based on these findings, we inferred that the differential expression of these novel miRNAs in different lambskin hair follicles plays an important role in the formation of lambskin patterns. Additionally, the expression of the rest of the seven miRNAs showed no significant differences among the large, medium, and small waves, and thus were not considered as candidate miRNAs. We preliminarily designated miRNA-143, miRNA-10a, let-7i, NW_004080184.1_6326, NW_004080165.1_8572, NW_004080181.1_3961, and NW_004080190.1_13733 as candidate miRNAs that regulate the development of Hu sheep hair follicles.

miRNAs in different cells form a comprehensive and multi-level network system through the interactions between signaling pathways and regulatory factors [49]. A large number of signaling pathways and regulatory molecules are involved in the steady-state maintenance of skin and hair follicle development, which include the Wnt and Akt signaling pathways, BMP, FGF, and Hox [50–51]. Studies have confirmed that miRNAs play important roles in the development of tissues and organs such as the brain, heart, liver, kidney, and skin [52–56]. Furthermore, miRNAs show specific expression profiles at different developmental stages and in various tissues. Recent research investigations have demonstrated that the expression profiles of miRNAs are tissue-specific. Previous studies have indicated that miRNAs are involved in the growth and development of the skin and hair follicles of various animals. Further research has also shown that miRNAs are differentially expressed at various stages of hair follicle growth. Moreover, miRNAs are important factors in the control of the growth and development of skin and hair follicles [4]. The present study showed that at the same miRNA expression level, the number of secondary follicles in small waves was higher than that in large waves, which was in agreement with our histological results. Combined with the study on the differential expression of miRNA-143 in large, medium, and small wave patterns, we inferred that miRNA -143 regulates Hu sheep hair follicle development.

Although no studies on the involvement of miRNA-10a and let-7i in sheep hair follicle development have been conducted to date, our research investigation has shown that at the same miRNA-10a expression level, the diameter of primary follicles in large waves was larger than that in small waves, and the diameter of secondary follicles in large waves was smaller than that in medium and small waves, and these findings were in agreement with our biopsy results. Furthermore, at the same let-7i expression level, the number of secondary follicles in large waves was lower than that in small waves, whereas the biopsy showed that these were similar. These discrepancies may be attributable to our small sample size as it is very difficult to finding full sibs of Hu sheep with different patterns. However, at the same let-7i expression level, the diameter of primary follicles in large waves was larger than that in medium waves, and these results was in agreement with our biopsy findings. The results of our correlation analysis basically agree with the findings of our corresponding biopsy examinations. In addition, in novel miRNAs, a correlation between the expression levels of NW_004080184.1_6326, NW_004080165.1_8572, NW_004080181.1_3961, NW_004080190.1_13733, miRNA-143, miRNA-10a, and let-7i and the diameter of secondary follicles in large and medium waves and the number of secondary follicles in small waves was observed, which coincided with our biopsy results. No significant differences in the expression levels of other miRNAs in the large, medium, and small waves were observed, although these showed correlations with related hair follicle indicators and thus were excluded as candidate miRNAs.

## Conclusions

In this study, 36 miRNAs were found to be differentially expressed, including 13 miRNAs among small and large waves, 20 miRNAs among small and medium waves, 14 miRNAs among medium and large waves. Gene ontology analysis indicated that they are involved in cell proliferation, differentiation, apoptosis, development, metabolization, and the immune response. The expression pattern of miR-143, miR-10a, miR-199p-3a, let-7c, let-7i, NW_004080189.1_7961, NW_004080184.1_6535, NW_004080184.1_6326, NW_004080165.1_8572, NW_004080166.1_9139, NW_004080181.1_3961, NW_004080174.1_726, NW_004080166.1_10417 and NW_004080190.1_13733 showed that they may participate in the formation of hair follicles and different flower pattern. The correlation analysis between these 14 miRNAs expression and histological properties of hair follicle in large, medium and small waves showed that miRNA-143, miRNA-10a, let-7i, NW_004080184.1_6326, NW_004080165.1_8572, NW_004080181.1_3961 and NW_004080190.1_13733 can be considered as the important candidate miRNA to Hu sheep lambskin hair follicle development. Our findings not only expanded the database on sheep miRNAs but also could be useful for future functional studies of miRNA involved in hair follicle development and different flower pattern formation in Hu sheep.

## Supporting information

S1 Table Relevant information of genes and primer sequences for Real Time PCR. (XLSX)

S2 Table Predicted targets of the significant differentially expressed miRNA in small waves and large waves. (XLSX)

S3 Table Predicted targets of the significant differentially expressed miRNA in medium waves and large waves. (XLSX)

S4 Table Predicted targets of the significant differentially expressed miRNA in small waves and medium waves. (XLSX)
